# Association of Selected State Policies and Requirements for Buprenorphine Treatment With Per Capita Months of Treatment

**DOI:** 10.1001/jamahealthforum.2023.1102

**Published:** 2023-05-26

**Authors:** Bradley D. Stein, Brendan K. Saloner, Olivia K. Golan, Barbara Andraka-Christou, Christina M. Andrews, Andrew W. Dick, Corey S. Davis, Flora Sheng, Adam J. Gordon

**Affiliations:** 1 RAND Corporation, Pittsburgh, Pennsylvania; 2 Johns Hopkins Bloomberg School of Public Health, Baltimore, Maryland; 3 School of Public Health, Georgia State University, Atlanta; 4 School of Global Health Management and Informatics, University of Central Florida, Orlando; 5 Arnold School of Public Health, University of South Carolina, Columbia; 6 RAND Corporation, Boston, Massachusetts; 7 Network for Public Health Law, Los Angeles, California; 8 RAND Corporation, Arlington, Virginia; 9 Program for Addiction Research, Clinical Care, Knowledge and Advocacy, Division of Epidemiology, Department of Internal Medicine, University of Utah School of Medicine, Salt Lake City; 10 Informatics, Decision-Enhancement, and Analytic Sciences Center, VA Salt Lake City Health Care System, Salt Lake City, Utah

## Abstract

**Importance:**

Expanding the use of buprenorphine for treating opioid use disorder is a critical component of the US response to the opioid crisis, but few studies have examined how state policies are associated with buprenorphine dispensing.

**Objective:**

To examine the association of 6 selected state policies with the rate of individuals receiving buprenorphine per 1000 county residents.

**Design, Setting, and Participants:**

This cross-sectional study used 2006 to 2018 US retail pharmacy claims data for individuals dispensed buprenorphine formulations indicated for treating opioid use disorder.

**Exposures:**

State implementation of policies requiring additional education for buprenorphine prescribers beyond waiver training, continuing medical education related to substance misuse and addiction, Medicaid coverage of buprenorphine, Medicaid expansion, mandatory prescriber use of prescription drug monitoring programs, and pain management clinic laws were examined.

**Main Outcomes and Measures:**

The main outcome was buprenorphine treatment months per 1000 county residents as measured using multivariable longitudinal models. Statistical analyses were conducted from September 1, 2021, through April 30, 2022, with revised analyses conducted through February 28, 2023.

**Results:**

The mean (SD) number of months of buprenorphine treatment per 1000 persons nationally increased steadily from 1.47 (0.04) in 2006 to 22.80 (0.55) in 2018. Requiring that buprenorphine prescribers receive additional education beyond that required to obtain the federal X-waiver was associated with significant increases in the number of months of buprenorphine treatment per 1000 population in the 5 years following implementation of the requirement (from 8.51 [95% CI, 2.36-14.64] months in year 1 to 14.43 [95% CI, 2.61-26.26] months in year 5). Requiring continuing medical education for physician licensure related to substance misuse or addiction was associated with significant increases in buprenorphine treatment per 1000 population in each of the 5 years following policy implementation (from 7.01 [95% CI, 3.17-10.86] months in the first year to 11.43 [95% CI, 0.61-22.25] months in the fifth year). None of the other policies examined was associated with a significant change in buprenorphine months of treatment per 1000 county residents.

**Conclusions and Relevance:**

In this cross-sectional study of US pharmacy claims, state-mandated educational requirements beyond the initial training required to prescribe buprenorphine were associated with increased buprenorphine use over time. The findings suggest requiring education for buprenorphine prescribers and training in substance use disorder treatment for all controlled substance prescribers as an actionable proposal for increasing buprenorphine use, ultimately serving more patients. No single policy lever can ensure adequate buprenorphine supply; however, policy maker attention to the benefits of enhancing clinician education and knowledge may help to expand buprenorphine access.

## Key Points

**Question:**

What is the association between selected state policies and buprenorphine dispensing?

**Findings:**

In this cross-sectional study using US county-level pharmacy claims data, educational requirements for buprenorphine prescribers beyond those required to obtain a waiver were associated with increased buprenorphine dispensing, as was continuing medical education related to substance misuse and addiction. Prescription drug monitoring programs, pain management clinic laws, and Medicaid policies had no association with buprenorphine dispensing.

**Meaning:**

These findings suggest that meaningful changes in access to buprenorphine may be achieved through greater attention to professional education and clinician knowledge.

## Teaser

This cross-sectional study uses US county-level pharmacy claims data to examine the association of selected state policies with rates of buprenorphine treatment.

## Introduction

The number of fatal opioid overdoses in the US continues to soar,^1^ and an estimated 1.6 million individuals in the country have an opioid use disorder (OUD).^2,3^ Medication treatment for opioid use disorder (MOUD)^4^ is considered the standard of care,^5^ improving quality of life and decreasing fatal overdose rates.^6,7,8,9^ Provision of MOUD in the US has grown substantially over the past 2 decades,^10,11^ driven largely by expansion in buprenorphine use.^12,13^ Despite this growth, less than one-third of individuals with OUD receive MOUD.^4,14^

Historically, federal law restricted buprenorphine prescribing for OUD to clinicians who obtained special permission from the federal government (commonly referred to as an X-waiver).^15,16^ Before the waiver requirement was removed at the end of 2022, most medical practitioners were required to receive 8 hours (physicians) or 24 hours (advanced practice clinicians) of specialized education to obtain one. Prior to the removal of the waiver requirement, the federal government and some state governments had enacted other policies that might influence the buprenorphine prescribing workforce, including increasing the number of patients waivered clinicians were permitted to treat,^17^ allowing advanced practice clinicians to prescribe buprenorphine for OUD,^17,18^ implementing mentoring and external facilitation intervention programs,^19,20,21^ requiring additional continuing medical education (CME) for buprenorphine prescribers,^22^ and requiring training in substance use disorder treatment for all controlled substance prescribers.^23^

The number of buprenorphine prescribers has grown substantially. However, many clinicians stop prescribing buprenorphine within 1 year of their first dispensed prescription,^24^ and most active prescribers treat few individuals.^25,26,27,28^ As a result, growth in the number of buprenorphine prescribers has not translated to comparable increases in buprenorphine use.

Multiple studies have examined how state policies affect buprenorphine access directly or affect proximal outcomes, such as the number of waivered clinicians. These studies have examined policies that affect the buprenorphine prescribing workforce directly as well as policies that could influence buprenorphine use through other mechanisms, such as financing, general clinician education, increasing demand for treatment, or Medicaid expansion.^29,30,31^ Other studies have examined policies requiring prescribers to use prescription drug monitoring programs (hereafter, mandatory PDMPs) and regulating pain management clinics.^32^ However, cross-study comparisons are challenging due to differences in measurement and sample selection. In addition, studies have often assessed effects of a single policy, although states often implement multiple policies that could influence buprenorphine access. To our knowledge, there are no national studies of the general population examining how state policies affect receipt of buprenorphine treatment, exploring how policy outcomes evolve over time, or disentangling how multiple policies implemented within a state could influence buprenorphine dispensing.^33^

To better understand how selected state policies are associated with buprenorphine dispensing, we examined the association of state policies with the rate of buprenorphine dispensing per 1000 county residents. Understanding such associations and the speed at which changes in buprenorphine use occur can inform policy maker efforts to enhance availability of buprenorphine treatment.

## Methods

### Data and Measures

In this cross-sectional study, we used deidentified pharmacy claims data from the IQVIA Real World Data–Longitudinal Prescriptions data set,^34^ which captures approximately 90% of prescriptions filled at retail pharmacies in all 50 states and the District of Columbia. We identified prescriptions for buprenorphine formulations with a US Food and Drug Administration–approved indication for OUD treatment dispensed from January 1, 2006, through December 31, 2018. Analyses were conducted from September 1, 2021, through April 30, 2022, with revised analyses conducted through February 28, 2023. The study adheres to the Strengthening the Reporting of Observational Studies in Epidemiology (STROBE) reporting guidelines for cross-sectional studies and was approved by the RAND institutional review board with a waiver of consent because the research used secondary data sets that did not contain information to identify or contact individuals.

We first defined buprenorphine treatment episodes. Episodes began with the first observed buprenorphine prescription dispensed with no buprenorphine prescription dispensed in the prior 90 days; the episode ended when the days’ supply from prior prescriptions was exhausted and there were no subsequent buprenorphine prescriptions within 30 days. If an individual was in an episode in any part of a calendar month, we considered that month an active treatment month for the individual. The prescriber responsible for most days’ supply in the episode was designated as the primary prescriber; we used the 5-digit Federal Information Process Standard code of the episode’s primary prescriber to determine the county in which the episode occurred. We aggregated active buprenorphine episodes for each calendar month at the county level to generate per capita rates of active treatment months for each county using data from the US Census Bureau.^35^ We smoothed the data with linear extrapolation and controlled for county-level factors previously found to be associated with buprenorphine dispensing or the number of waivered buprenorphine clinicians.

### County Measures

Based on Rural-Urban Continuum Codes (RUCC) from the Area Health Resources Files, we classified counties as metropolitan (RUCC 1, 2, or 3) or rural (RUCC 4-9).^36^ We calculated county drug overdose rates as the per capita rate of overdose deaths using the restricted multiple cause of death mortality file from the Centers for Disease Control and Prevention^37^ and, given prior studies documenting differences in buprenorphine distribution by percentage of non-White county residents,^38^ from the American Community Survey.^39^

### Policy Measures

Our analysis included 6 state-level policies (Table 1). Our choice of policies was a function of those that have been found in the literature to be associated with buprenorphine prescribing or waivered prescribers (Medicaid coverage, Medicaid expansion, mandatory PDMPs, pain management clinic regulations); policies that conceptually could influence knowledge and behaviors related to substance use disorder and OUD treatment, even if not previously examined empirically; and our ability to obtain policy information for all or almost all states over the study period.

**Table 1.** Description of Selected State Policies

State policy	Description
Mandatory PDMP	A state has an operational PDMP and mandates that prescribers in that state check the PDMP before prescribing opioids for a patient. States that only mandate use by pharmacists or dispensers are not included in this definition.^40^
Pain management clinic laws	Laws that identify specific clinics focused on pain management, regulate personnel and oversight, and regulate the prescribing practices of clinicians working in those clinics.^41^
Additional education for buprenorphine prescribers	Policies requiring additional education for buprenorphine prescribers in addition to the education required to obtain an X-waiver.^22^
CME related to substance misuse and addiction for licensure	Policies requiring CME related to substance misuse and addiction for licensure.
Medicaid coverage of buprenorphine	The state Medicaid agency covers buprenorphine for the treatment of opioid use disorder.^42^
Medicaid expansion	The state expanded Medicaid coverage under the Affordable Care Act.^43^

Abbreviations: CME, continuing medical education; PDMP, prescription drug monitoring program.

We identified the month in which a state implemented mandatory PDMPs from the OPTIC-Vetted Policy Data Set,^40^ which reviews laws and documents decisions made in creating data sets. We obtained information on the month a state implemented a pain management clinic law from the Prescription Drug Abuse Policy System, which conducts a legal review.^41^ We searched the Westlaw legal database to identify the month in which a state required additional education for buprenorphine prescribers beyond the X-waiver requirement or required CME related to substance misuse and addiction for licensure.^22,44^ Information on Medicaid coverage of buprenorphine was derived from surveys of state Medicaid officials, including 1 in 2013 to 2014^42^ and another in 2020 (unpublished data). We obtained information regarding state Medicaid expansion under the Affordable Care Act from the Kaiser Family Foundation.^43^ Medicaid covers approximately 40% of all OUD treatment.^45^ Changes in Medicaid policy have been associated with changes in the use of buprenorphine among Medicaid enrollees and in other patient populations.^46^ The Figure shows the year in which states implemented each policy.

**Figure.**
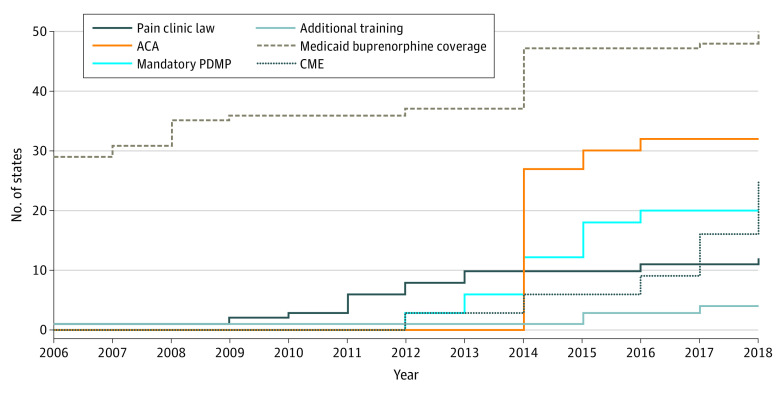
Month and Year of Implementation of Selected State Policies, January 2006 to December 2018 ACA indicates the Affordable Care Act; CME, continuing medical education; PDMP, prescription drug monitoring program.

### Statistical Analysis

To examine the association of selected state policies with county-level per capita rates of active buprenorphine treatment, we specified a multivariable event-time model to assess multiple policies simultaneously, adapting work by Borusyak et al^47^ and Goodman-Bacon.^48^ Our model controls for county characteristics and for other state policies that may be associated with rates of buprenorphine treatment. States implemented different policies at different times. Therefore, for each county-month, we defined event-time indicators as the months since (or until) each policy was implemented (the event), with the exception of Medicaid coverage of buprenorphine, for which we only had information on the first year each state had coverage. Therefore we used the midpoint (July 1) of the first year the state had Medicaid coverage as the date on which the policy was implemented. We included event-time indicators for months before policy adoption to identify endogenous policy adoption (ie, policies adopted in response to worsening conditions, such as increased need for treatment) or anticipatory effects (eg, policy effects on outcomes before policy implementation, possibly as a result of preparing for the policy).

To distinguish policy effects from secular trends, we included calendar-time indicators as main effects. To allow for states that adopted different policies to have different secular trends, we included calendar-time interactions with groups of states that ever adopted each policy.

Using county-month as the unit of observation, we specified ordinary least squares models of per capita rates of active buprenorphine treatment episodes as a function of policy event-time indicators, calendar-time indicators, calendar-time indicators interaction with state policy groups, and county characteristics. We also included state fixed effects and calculated Huber-White robust SEs clustered at the state level. Finally, we applied weights that were proportional to county population and summed to 1 in each state-month. Thus, observations were representative of states, and the models generated state average policy associations.

In the eMethods in Supplement 1, we detail how we determined the best specification for prepolicy implementation association. We first examined the full set of event-time indicators before the policy was implemented. Finding no significant associations before 1 year, we focused on the 12 months before policy implementation. We found evidence of positive associations during that period with 2 of the policies (requirements for additional education of buprenorphine prescribers and CME); we used the period 13 months before implementation as the referent to address anticipatory effects. For the remaining 4 policies, we restricted the event-time indicators for the last month before policy implementation to 0. We present the estimates and 95% CIs of the change in total treatment months per capita for each 1-year period postpolicy adoption from the first to the fifth year. The statistical analysis was performed using SAS, version 9.4 software (SAS Institute Inc).

## Results

The mean (SD) number of months of buprenorphine treatment per 1000 persons nationally increased steadily from 1.47 (0.04) in 2006 to 22.80 (0.55) in 2018 (Table 2). Controlling for county characteristics and other policies, we found that required education of buprenorphine prescribers beyond the waiver requirement was associated with significant increases in the number of months of buprenorphine treatment per capita in the year following policy implementation (an increase of 8.51 treatment months per 1000 population in the first year; 95% CI, 2.36-14.64 months). The magnitude of the association increased in each of the 5 years following implementation to 14.43 months of treatment per 1000 population in the fifth year (95% CI, 2.61-26.26) (Table 3). Requiring CME for physician licensure related to substance misuse and addiction was also associated with a significant increase in buprenorphine use in each of the 5 years following implementation (an increase of 7.01 treatment months per 1000 population in the first year; 95% CI, 3.17-10.86 months), growing to 11.43 treatment months per 1000 population in the fifth year (95% CI, 0.61-22.25 months). In contrast, after controlling for county characteristics and other policies implemented, we found no association between buprenorphine months of treatment per 1000 county residents and Medicaid expansion, Medicaid coverage of buprenorphine, pain management clinic regulations, or mandatory PDMPs.

**Table 2.** Buprenorphine Months of Treatment per 1000 Persons in the US, per Year, 2006-2018

Variable	Mean (SD) months
Year	
2006	1.47 (0.04)
2007	2.85 (0.07)
2008	4.71 (0.10)
2009	6.38 (0.13)
2010	7.43 (0.15)
2011	8.92 (0.18)
2012	10.86 (0.22)
2013	12.82 (0.26)
2014	14.47 (0.32)
2015	16.09 (0.37)
2016	17.55 (0.41)
2017	19.95 (0.48)
2018	22.80 (0.55)
County urbanicity	
Urban	11.68 (0.45)
Rural	8.70 (0.20)
County fatal overdose rate, quartile	
1 (Lowest)	3.25 (0.09)
2	6.84 (0.21)
3	10.56 (0.33)
4 (Highest)	19.80 (0.46)
County percentage of non-White residents, quartile^a^	
1 (Lowest)	14.01 (0.29)
2	13.23 (0.37)
3	13.05 (0.31)
4 (Highest)	9.33 (0.29)

^a^ Calculated based on race and ethnicity categories from the American Community Survey.^39^

**Table 3.** Annual Change in Buprenorphine Treatment Months per 1000 County Residents in 5 Years Following Policy Implementation^a^

Year since policy adoption	Effect (95% CI)					
	Required additional education for buprenorphine prescribers	Required CME related to substance misuse and addiction	Medicaid coverage of buprenorphine	Medicaid expansion	Mandatory PDMP	Pain management clinic regulations
First	8.51 (2.36 to 14.64)	7.01 (3.17 to 10.86)	−1.73 (−4.33 to 0.88)	0.09 (−6.22 to 6.40)	−2.62 (−8.10 to 2.85)	2.20 (−2.17 to 6.57)
Second	12.54 (4.63 to 20.44)	9.97 (3.46 to 16.48)	−1.82 (−4.78 to 1.14)	−0.72 (−9.37 to 7.93)	−2.10 (−10.92 to 6.73)	2.59 (−3.08 to 8.26)
Third	12.85 (2.05 to 23.66)	8.95 (1.95 to 15.95)	−1.78 (−5.06 to 1.50)	−0.80 (−11.85 to 10.25)	−2.42 (−15.57 to 10.72)	3.61 (−3.39 to 10.61)
Fourth	13.93 (0.25 to 27.62)	11.62 (2.43 to 20.8)	−1.67 (−5.27 to 1.93)	−2.45 (−16.28 to 11.39)	−0.92 (−18.98 to 17.14)	4.70 (−3.84 to 13.24)
Fifth	14.43 (2.61 to 26.26)	11.43 (0.61 to 22.25)	−1.82 (−6.01 to 2.38)	−7.89 (−24.80 to 9.02)	5.12 (−18.90 to 29.13)	5.30 (−4.46 to 15.06)

Abbreviations: CME, continuing medical education; PDMP, prescription drug monitoring program.

^a^ The point estimate is change in buprenorphine treatment months per 1000 county residents.

## Discussion

In this cross-sectional analysis of how multiple state laws were associated with buprenorphine dispensing outcomes, we found that requiring additional education of clinicians who prescribe buprenorphine beyond waiver requirements was associated with substantial increases in buprenorphine treatment per capita. To put this finding into context, in a county with 200 000 residents, by the fourth year after policy implementation, an increase of more than 10 additional patient-months per 1000 residents would result in an additional 333 individuals receiving buprenorphine treatment, assuming an average treatment episode of 6 months.

Multiple studies have found that clinician apprehensions about being sufficiently knowledgeable regarding buprenorphine treatment for OUD is a barrier to prescribing buprenorphine,^49,50,51,52,53^ and providing clinicians with additional education, training, or mentorship beyond the waiver requirement has been associated with increased buprenorphine treatment.^54,55^ Given concern that requiring training just to obtain an X-waiver may have dissuaded clinicians from prescribing buprenorphine, it may seem counterintuitive that requiring additional training is associated with more buprenorphine prescribing. However, lack of confidence in treating patients and the clinical complexity of patients, issues that are commonly reported barriers to buprenorphine prescribing,^49,51,53,56,57,58,59^ may be addressed in ongoing training. Additional training may increase the buprenorphine-prescribing clinicians’ confidence, enhancing their willingness to treat more patients or to keep patients in treatment longer. Our finding is consistent with studies showing that educational efforts, albeit not mandated by state policy, have been associated with increased buprenorphine prescribing.^54,55,60,61^ Furthermore, recent research finding that federal relaxation of training requirements to obtain a waiver did not substantially increase the number of clinicians obtaining waivers suggests that the barrier presented by training may not be as great as many feared.^16,62^

We also found a significant association between buprenorphine dispensing and laws requiring CME in substance misuse and addiction for physician relicensure. Concerns have been expressed about the adequacy of physician education regarding substance use disorders^63^ and primary care physician understanding of MOUD.^64^ Continuing medical education focused on substance use disorders and the treatment of such disorders may help to mitigate such knowledge gaps. Clinicians already confident or interested in substance use disorder treatment might self-select into voluntary CME courses. However, mandatory CME courses may alleviate concerns about addiction treatment or increase interest in addiction treatment among clinicians who would not otherwise choose such courses, thus potentially increasing the pool of buprenorphine prescribers. The Consolidated Appropriations Act of 2023 eliminated the requirement for waivers but requires the Substance Abuse and Mental Health Services Administration to establish training requirements related to substance misuse and addiction treatment for most clinicians obtaining a Drug Enforcement Administration license to prescribe controlled substances.^65^ Such training could help allay self-efficacy concerns among clinicians interested in prescribing buprenorphine for OUD, potentially increasing buprenorphine prescribing rates.

Even if CMEs do not increase the local pool of buprenorphine prescribers, they may increase awareness of buprenorphine treatment efficacy, leading clinicians to support patients’ ongoing engagement in treatment and motivating nonprescribers encountering a patient with OUD to make an appropriate referral. Additional research is needed to better appreciate how required education is associated with buprenorphine treatment. However, our findings suggest that policies requiring additional education regarding substance misuse and substance use disorder treatment more generally may contribute to increased buprenorphine use.

Medicaid remains the predominant payer for OUD treatment.^66^ Studies have found an association between Medicaid expansion and Medicaid coverage of buprenorphine and increases in the number of buprenorphine-waivered prescribers, buprenorphine receipt among Medicaid-enrollees in the year after expansion, more dispensed buprenorphine prescriptions written by nurse practitioners and physician assistants, and fewer overdose deaths.^29,30,31,67,68^ In our analyses, we did not find Medicaid coverage for buprenorphine treatment or Medicaid expansion to be associated with buprenorphine treatment per capita. States that expanded Medicaid may differ substantially from those that did not with respect to other policies related to OUD treatment. We examined the outcomes of multiple relevant policies in a state simultaneously. Thus, our findings may be influenced by the fact that our analysis accounts for additional state policies likely to influence buprenorphine use. It is also possible that compared with Medicaid expansion states, states that did not expand Medicaid supported buprenorphine treatment with resources from short-term funding mechanisms, such as State Opioid Response grants and State Targeted Response grants established by the 21st Century Cures Act.^69,70^ In addition, research has shown that increased buprenorphine treatment after Medicaid expansion was offset by decreases in treatment in other populations,^46^ a result consistent with other studies that found little effect of Medicaid expansion on overall rates of substance use disorder treatment in the general population.^71,72^ Furthermore, multiple studies have found that many buprenorphine prescribers do not accept Medicaid.^73,74^ Thus, our findings highlight the essential contribution of state policy efforts to expand access to buprenorphine treatment beyond Medicaid.

We also found no association with policies regulating pain management clinics or mandatory PDMP policies. Although pain specialists represent approximately 8% of buprenorphine prescribers^25^ and PDMPs could facilitate prescriber identification of individuals potentially misusing opioids, it seems likely that pain management clinics and PDMPs are more likely to influence opioid analgesic prescribing than to directly influence buprenorphine dispensing.

### Limitations

Our findings must be considered within the context of study limitations. We restricted our analyses to buprenorphine formulations indicated for OUD treatment, but we cannot identify off-label prescriptions of those formulations. We do not know the clinical status of patients to whom buprenorphine was dispensed or the clinical setting in which prescriptions were written. We also lack information on patient race and ethnicity, factors associated with disparities in receiving buprenorphine.^75^ We do not know whether our results generalize to prescriptions dispensed from pharmacies not captured in the IQVIA data or to prescriptions written but not dispensed.

Prior to the elimination of the waiver, in recent years, multiple federal policies designed to increase the availability of buprenorphine had been implemented, including increasing patient limits for waivered prescribers,^76^ allowing clinicians other than physicians to obtain waivers or to manage the treatment of patients with OUD through collaborative relationships,^77,78^ and permitting some clinicians to obtain a waiver without training.^15^ These efforts have substantially increased the number of buprenorphine-waivered clinicians, especially among nonphysician prescribers such as nurse practitioners and physician assistants.^76^ Buprenorphine dispensing could also be associated with other state policies, such as those addressing counseling requirements, prior authorization, clinician monitoring of patients, prescribing of buprenorphine by nonphysicians, state methadone policies, and state Section 1115 waivers of the Institutions for Medical Disease exclusion allowing use of Medicaid funds to treat substance use disorders in larger inpatient facilities.^79,80,81^

Unfortunately, we do not have the data required to examine the outcomes of such policies across all states and the entire study period, nor do we have sufficient current prescribing data to examine the outcomes associated with more recent policy changes. Our analytic approach does not allow us to examine US guidelines and nonpolicy efforts designed to increase buprenorphine prescribing, and we do not have systematic information about other efforts that may occur locally, regionally, or within health systems, such as the Veterans Administration.^20,21,82^ Our data also do not allow us to examine the outcomes of policies implemented during the COVID-19 pandemic that might be associated with dispensing of buprenorphine to treat OUD.

## Conclusions

The findings from this cross-sectional study make an important contribution to the literature. Improving access to buprenorphine is essential to treating more patients with OUD effectively and decreasing overdoses and other OUD-related harms. Our study points to required education in substance misuse and substance use disorders as an actionable proposal for increasing buprenorphine use and ultimately serving more patients. This finding is important because the proposal is feasible to implement: CME and other requirements can be directly modified by state medical boards, are a relatively low burden for clinicians to complete, and can be targeted to groups of nonprescribing clinicians who serve patients who might benefit from buprenorphine. Thus, this model can be replicated in states currently without similar CME requirements. Continuing medical education is also a potential target for federal action, and state policies that would require controlled substance prescribers to have some basic training in the treatment of substance use disorders are in some ways similar to recently enacted federal legislation.^23^

No single policy lever can boost buprenorphine treatment to its optimal scale. However, achievable and meaningful changes in access may be achieved through greater attention to professional education and clinician knowledge. Moving forward, additional high-quality longitudinal policy data sets are needed to facilitate evaluation of policies intended to increase buprenorphine prescribing.

## Notes

Supplement 1. **eMethods.** Detailed Description of Methods

Supplement 2.
Data Sharing Statement
