# Supplementary material for: Association of Selected State Policies and Requirements for Buprenorphine Treatment With Per Capita Months of Treatment
Source: JAMA Health Forum. 2023 May 26;4(5):e231102. doi: 10.1001/jamahealthforum.2023.1102 (PMC10220518; doi:10.1001/jamahealthforum.2023.1102)
Supplement: Supplement 1. — eMethods. Detailed Description of Methods [file jamahealthforum-e231102-s001.pdf]

Supplemental Online Content

Stein BD, Saloner BK, Golan OK, et al. Association of selected state policies and requirements for buprenorphine treatment with per capita months of treatment. *JAMA Health Forum*. 2023;4(5):e231102. doi:10.1001/jamahealthforum.2023.1102

eMethods. Detailed Description of Methods

eReferences

This supplemental material has been provided by the authors to give readers additional information about their work

© 2023 Stein BD et al. *JAMA Health Forum.*

eMethods. Detailed Description of Methods

Adapting methods from Goodman-Bacon and Borusyak,[1,2] we specified a longitudinal model that relies on staggered policy adoption across states to identify policy effects. Models such as these are referred to as event-time models, where event-time is defined as the time until or the time since policy adoption. Thus, our models have two time metrics: calendar time, which identifies secular trends that may be common across states, and event-time, which identifies the timing of an outcome relative to the timing of policy adoption and is identified separately from calendar time because of the staggered adoption of policies across states. Among event-time models, ours is also referred to as a 2-way fixed effect model because we specify calendar time as a vector of monthly fixed-effect indicators and we include state fixed-effects, allowing for across state comparisons of within-state pre- versus post-policy intervention differences (or difference-in-differences).

We chose to examine multiple policies simultaneously, as experts have noted not doing so is an important limitation of many opioid policy analyses.[3] The policy wheels, Appendix Figure 1 below, illustrate the pattern of adoption of policies across states. Furthermore, an empirical examination of the policies we examine demonstrate a substantively important level of correlation among the policies, as shown in Appendix Table 1.

eFigure 1. Policy Wheels Illustrating State Adoption of Policies Examined Over Time

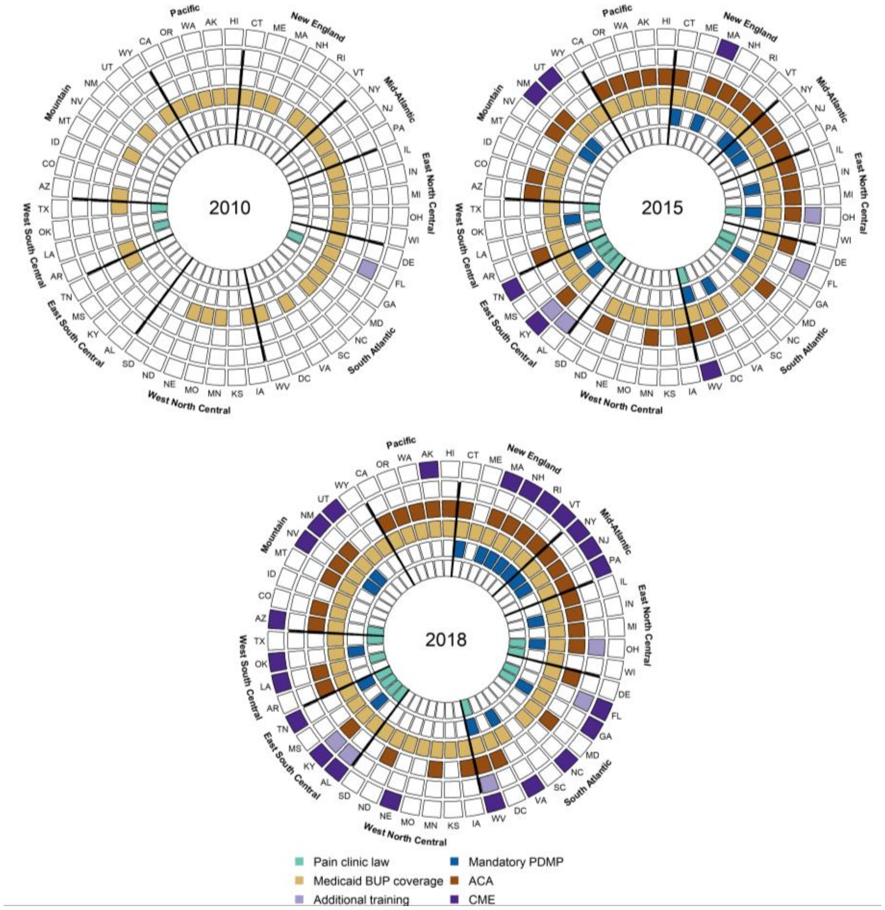

© 2023 Stein BD et al. *JAMA Health Forum.*

Appendix Table 1: Correlation Matrix of State Policies, Weighted Estimates

Pearson Correlation Coefficients, N = 489984<br>Prob > |r| under H0: Rho=0						
	Required CME related to substance misuse and addiction	Required additional education for buprenorphine prescribers	Mandatory PDMP	Pain management clinic regulations	Medicaid expansion	Medicaid coverage of buprenorphine
Required CME related to substance misuse and addiction	1.00000					
Required additional education for buprenorphine prescribers	0.12928<br><.0001	1.00000				
Mandatory PDMP	0.58757<br><.0001	0.13416<br><.0001	1.00000			
Pain management clinic regulations	0.21919<br><.0001	0.42722<br><.0001	0.24855<br><.0001	1.00000		
Medicaid expansion	0.27619<br><.0001	0.06116<br><.0001	0.39980<br><.0001	-0.00245<br>0.0861	1.00000	
Medicaid coverage of buprenorphine	0.15070<br><.0001	0.04799<br><.0001	0.17055<br><.0001	0.04382<br><.0001	0.27928<br><.0001	1.00000

Our general approach was to begin by specifying a full set of monthly event-time indicators, starting at the beginning of the study period and placing no limits on time-until policy adoption, and carrying through the end of the study period with no limits on time-since policy adoption. This allowed us to examine evidence of pre-policy adoption trends in event-time with limited restrictions. Following Goodman-Bacon and Borusyak,[1,2] we restricted several arbitrarily-selected pre-policy adoption event-time indicators to zero to identify event-time policy effects separately from calendar time effects. Without the restrictions, the two time-metrics would only be identified up to a linear trend, which could be generated by either metric. After rejecting long-term trends, we focused on the year before implementation. We set all event-time indicators prior to the 12th pre-implementation month to zero, and we further scrutinized the period 12-months before policy implementation, the period most likely to show evidence of anticipatory effects should they arise. If we found evidence of anticipatory effects for a policy, we retained that policy's even-time indicators for the 12-month before policy implementation. If we found no evidence of anticipation, we further restricted the last event-time month prior to policy implementation to zero, and we retained the remaining 11 pre-implementation event-time indicators to show the pre-implementation trends. Appendix Table 2 provides our regression results including the year prior to policy implementation, illustrating the anticipatory effects we identified.

© 2023 Stein BD et al. *JAMA Health Forum.*

Appendix Table 2: Annual change in buprenorphine treatment months per 1,000 county residents

Year since policy adoption	Required additional education for buprenorphine prescribers		Required CME related to substance misuse and addiction		Medicaid coverage of buprenorphine		Medicaid expansion		Mandatory PDMP		Pain management clinic regulations	
	Effect	95% CI	Effect	95% CI	Effect	95% CI	Effect	95% CI	Effect	95% CI	Effect	95% CI
Prior year	5.55	1.29-9.8	4.16	2.0-6.31	-0.66	-2.23-0.91	0.90	-1.78-3.58	-1.15	-3.77-1.48	0.93	-1.36-3.22
1st year	8.51	2.37-14.64	7.01	3.17-10.86	-1.73	-4.33-0.88	0.09	-6.22-6.4	-2.62	-8.1-2.85	2.20	-2.17-6.57
2nd year	12.54	4.63-20.44	9.97	3.46-16.48	-1.82	-4.78-1.14	-0.72	-9.37-7.93	-2.10	-10.92-6.73	2.59	-3.08-8.26
3rd year	12.85	2.05-23.66	8.95	1.95-15.95	-1.78	-5.06-1.5	-0.80	-11.85-10.25	-2.42	-15.57-10.72	3.61	-3.39-10.61
4th year	13.93	0.25-27.62	11.62	2.43-20.8	-1.67	-5.27-1.93	-2.45	-16.28-11.39	-0.92	-18.98-17.14	4.70	-3.84-13.24
5th year	14.43	2.61-26.26	11.43	0.61-22.25	-1.82	-6.01-2.38	-7.89	-24.8-9.02	5.12	-18.9-29.13	5.30	-4.46-15.06

- The point estimate is change in buprenorphine treatment months per 1000 county residents

© 2023 Stein BD et al. *JAMA Health Forum.*

We start here by describing a single policy model and then generalizing to the multiple policy model we estimated.

Model with 1 policy.

Let *t* be a calendar time index and ET be an event time index defined as *t* less the month of policy implementation. With data structured so that the county-month was the unit of analysis, we express the single policy model as:

$$Y_{ist} = \beta_0 + \beta_1 X_{ist} + \sum_t \left(\beta_{2,t} T_t + \beta_{3,t}(G_s * T_t)\right) + \sum_{ET_{s,t}} \left(\beta_{4,ET} \tau_{ET_{s,t}}\right) + FE_s + \varepsilon_{ist}, \qquad (1)$$

where Y_{ist} is total monthly number in treatment per capita for which *i*, *s*, and *t* index county, state, and calendar month; *X* is a vector of covariates, T_t is a calendar month indicator for month *t*, *G* is a treatment group indicator for all states that ever implemented the policy; $\tau_{ET_{s,t}}$ is an indicator for event-time in state *s* at time *t* ($ET_{s,t}$); FE_s is a vector of state fixed effects; and ε_{ist} is an error term. β_2 and β_3 are parameter vectors for all *t*, and β_4 is a parameter vector for all ET, and therefore identifies policy associations both before and after policy implementation.

Identification of ET effects: In these models, even with rich data, *ET* is identified separately from $G_s * T_t$ only up to a common linear trend. Our general approach followed the suggestions of Goodman-Bacon (2021) and Borusyak and colleagues (2022).[1,2] First, we estimated the model with no restrictions on ET and examined the pattern of coefficients. Second, we restricted a set of arbitrarily chosen coefficients of ET<0 (we selected ET=-25, ET=-31, ET=-37, …) to be zero, which required any linear trend to be absorbed by calendar time. In the absence of evidence of long-term pre-implementation trends, we further restricted all ET<-12 to zero and scrutinized the last 12 months before policy implementation.

Considerations

Potential bias in long-duration effects: Variation in the timing of policy implementation across states requires that the horizon with which effects are identified will vary by state. Thus, while it is necessary to admit long-duration effects to ensure that bias is not introduced among other model parameters, their estimates may not be reliable as the number of states supporting them decline. Therefore, although we specified ET monthly through the end of the study period, we test for effects and display results only through 5 years.[1]

Focus on average state effects versus average person effects: These models are almost always estimated at the individual level, producing estimated effects for the average person. The models answer questions like: *What did these policies do to the U.S. population?* To answer questions such as *What was the average (across states) effect of a policy that was implemented in 10 states?* the data should be weighted to make the contribution of large and small states the same. Because our unit of analysis is the county-month, we want a county's weight to be proportional to its population within the state-month, but we want every state-month to have equal weight. The straight-forward approach is to create weights as

$$W_{ist} = N_{ist}/N_{st},$$

where N_{ist} is the population of county *i,* state *s,* at time *t*; and N_{st} is the population of state *s* at time *t.* The sum of the weights for each state-month is 1, but within states the weights are proportional to the

© 2023 Stein BD et al. *JAMA Health Forum.*

size of the county's population. This also addresses concerns about negative weights in regression estimates of heterogenous policy effects over time since policy implementation.[1,2]

Model with K policies

Generalizing to multiple policies from (1) generates a large number of parameters and can only work in applications with very rich data. At a minimum, there must be adequate variation in the adoption of policies across states and over time (illustrated above in the policy wheels in Appendix Figure 1 and Appendix Figure 2, which shows both the policy implementation by state by year, using a modification of the figure in the original manuscript which reviewers suggested we revise, as well as now including an illustration of the treatment trend by state). Consider the direct generalization of (1):

$$Y_{ist} = \beta_0 + \beta_1 X_{ist} + \sum_t \left[\beta_{2,t} T_t + \sum_k \beta_{3,t}^k \left(G_s^k * T_t\right)\right] + \sum_k \sum_{ET_{s,t}^k} \left(\beta_{4,ET}^k ET_{s,t}^k\right) \; + FE_s + \varepsilon_{ist} \qquad (2)$$

where k indexes policies. This model calculates the calendar time effects (β_3) for each of the K state-policy groups (G) conditional on ET estimates that may be active during the pre- or post-adoption periods in other state-policy groups. Identifying these effects requires adequate variation (both across states and over time) in each state-policy group and across policies. If in any month, the states in $G_s^{k=a}$ are the same as in $G_s^{k=b}$, the parameters are not identified separately; however, their combined deviation from the main effects is identified, which is sufficient.

We estimated equation (2) and, using the approach described above for the single policy model, we examined the coefficients of each policy for evidence of anticipation. In this application, we found evidence of anticipatory effects for two policies (additional training and continuing medical education). For the remaining four policies, we further restricted the event-time indicator just prior to policy adoption (ET=-1) to zero.

© 2023 Stein BD et al. *JAMA Health Forum.*

Appendix Figure 2: Year of policy implementation by state January 2006 – December 2018, with trends in average treatment months per 1000 county residents for 50 states and DC (weighted by county population)

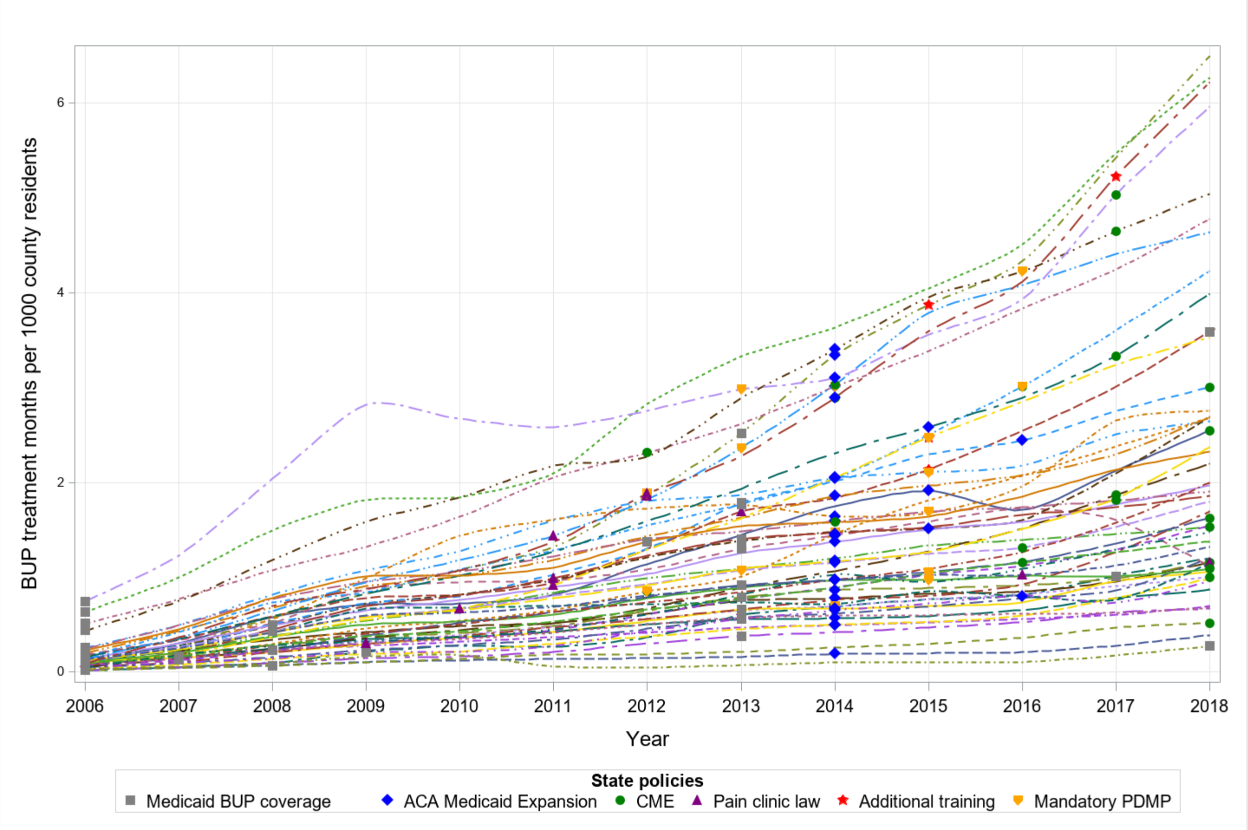

We characterized the effect sizes and statistical significance of the effects in annual segments by identifying the areas between the estimated ET function and the counterfactual (horizontal line at zero) for each 12-month period after policy implementation. We also provide graphs of the ET estimates by month for each policy in Appendix Figures 3a – 3f.

© 2023 Stein BD et al. *JAMA Health Forum.*

Appendix Figure 3a- Continuing Medical Education

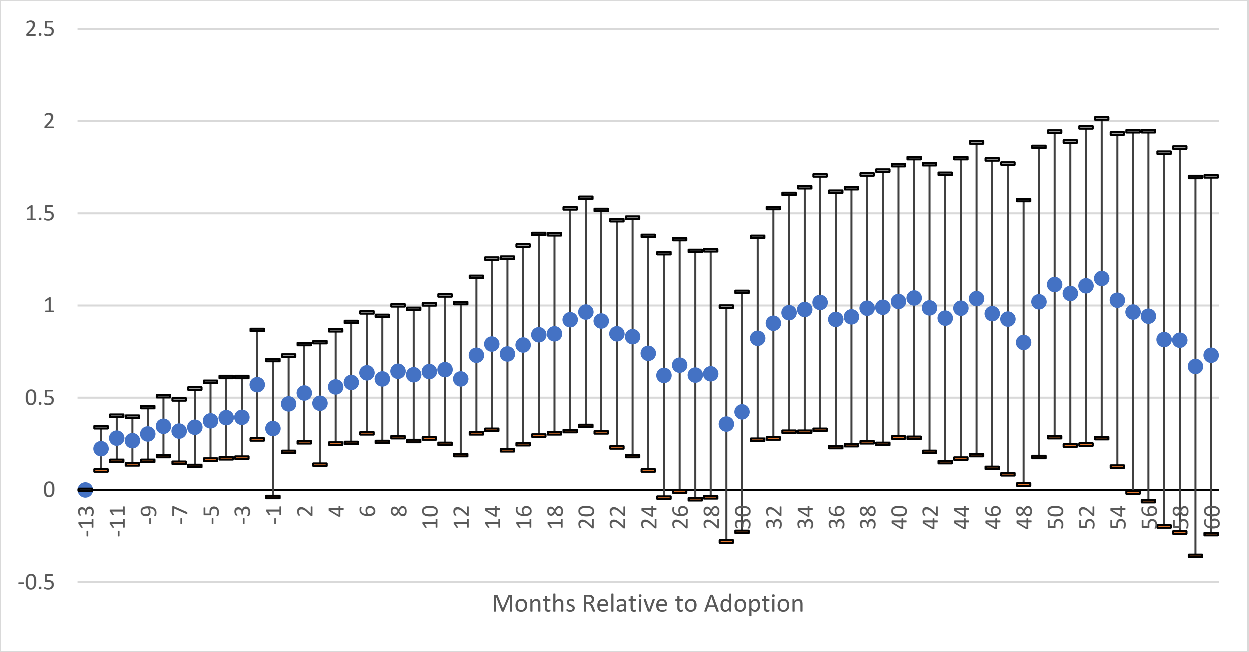

Appendix Figure 3b- Affordable Care Act Medicaid Expansion

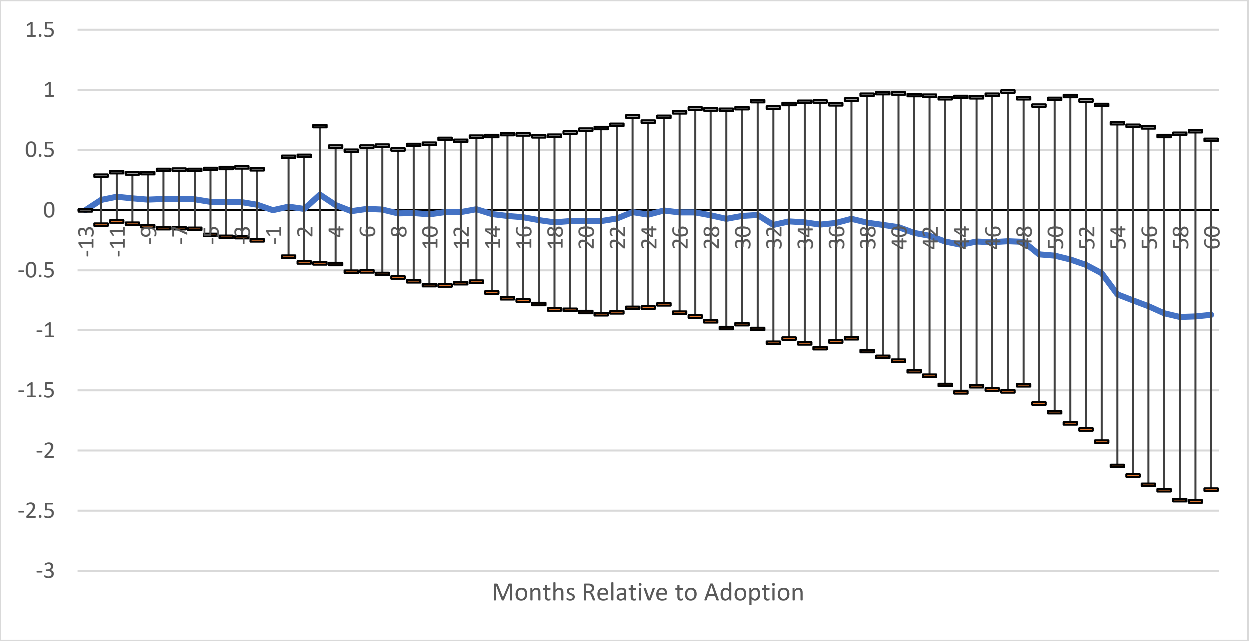

© 2023 Stein BD et al. *JAMA Health Forum.*

Appendix Figure 3c- Pain Management Clinic Regulations

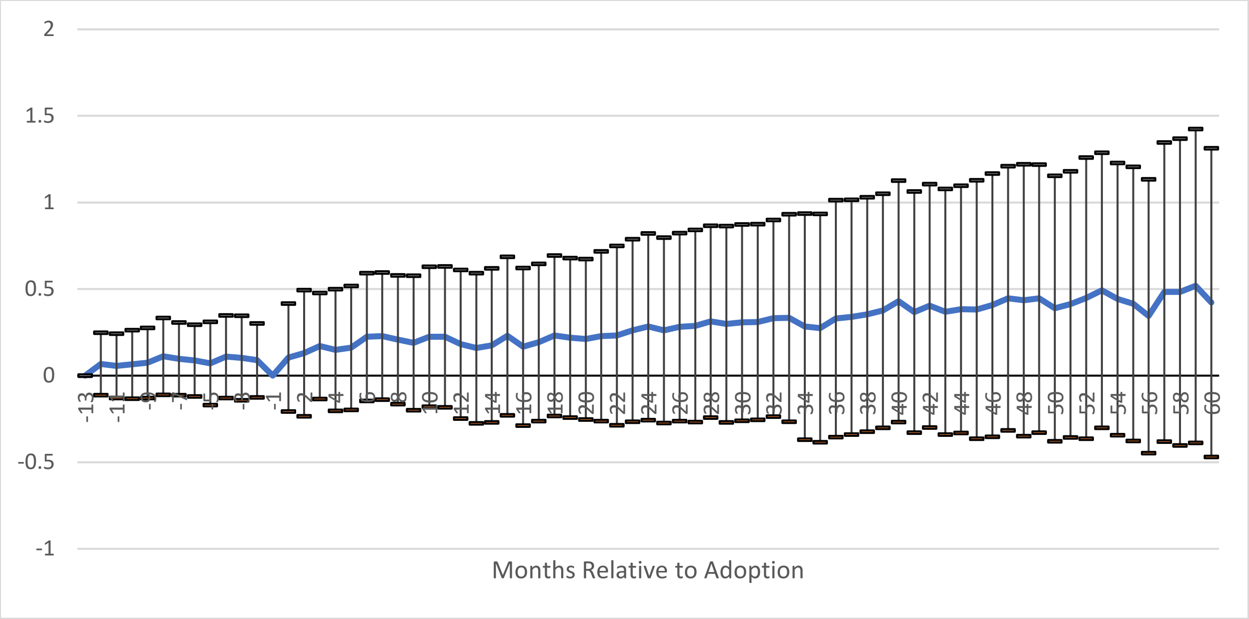

Appendix Figure 3d- Additional Education for Buprenorphine Prescribers

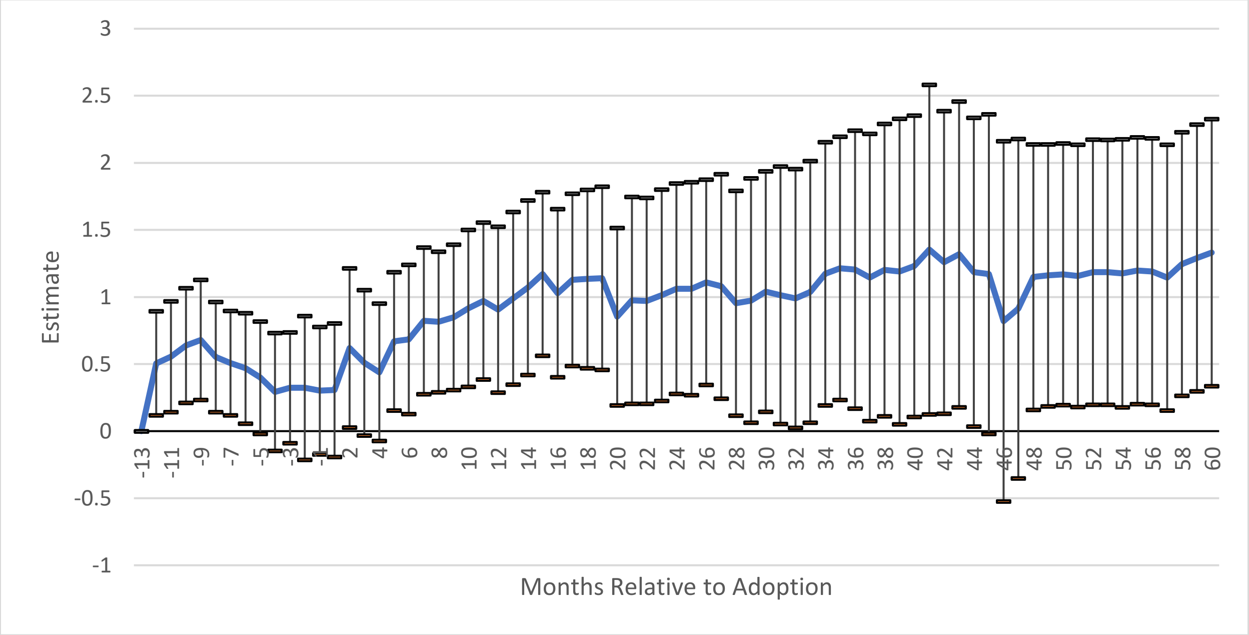

© 2023 Stein BD et al. *JAMA Health Forum.*

Appendix Figure 3e- Mandatory PDMP

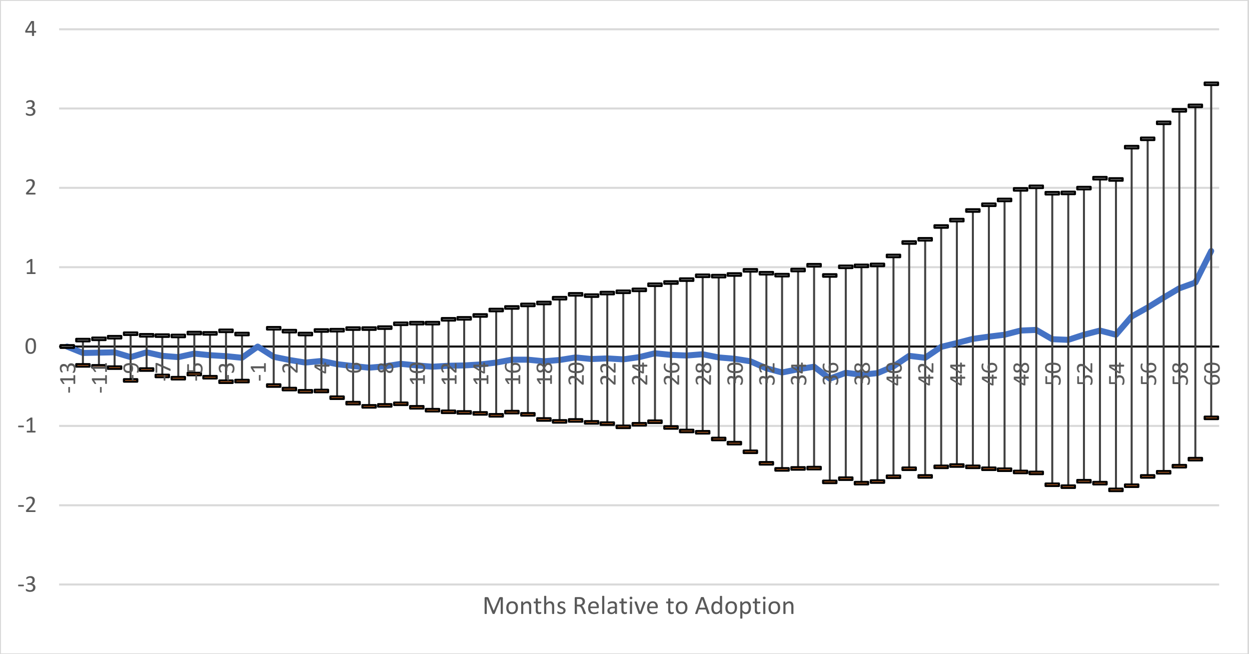

Appendix Figure 3f- Medicaid Coverage of Buprenorphine

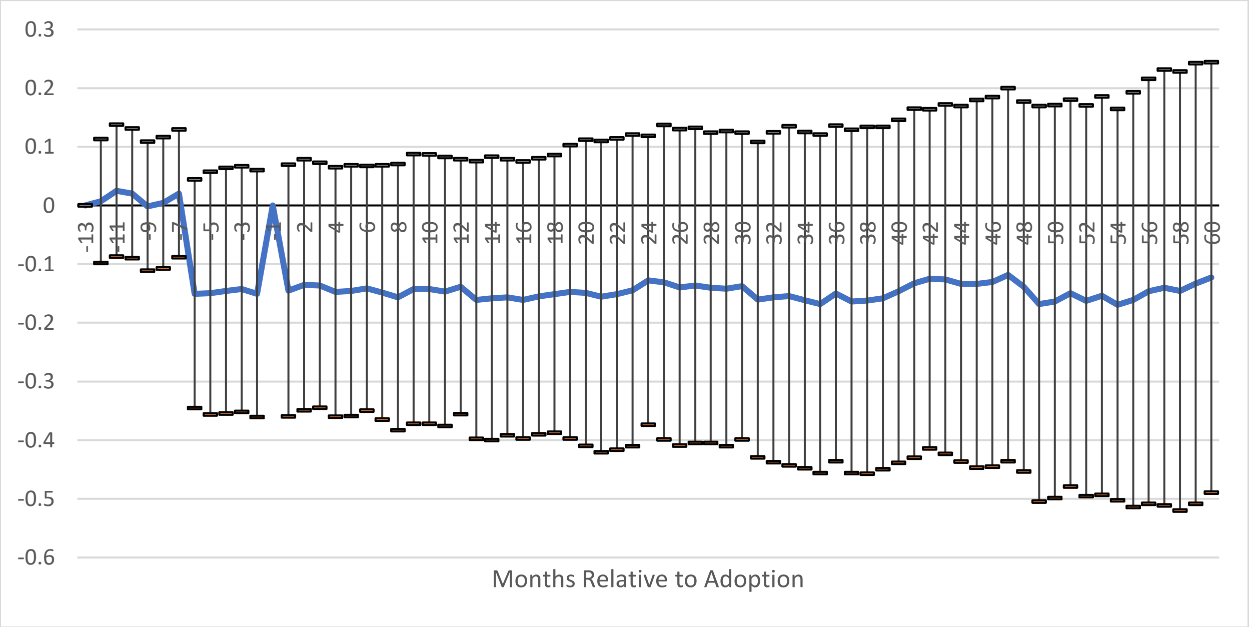

© 2023 Stein BD et al. *JAMA Health Forum.*

Limitations

There have been many recent advances in event-study models. Although we rely heavily on Goodman-Bacon and Borusyak,[1,2] we have not attempted to adapt the most recently suggested methods, which were developed for single policy models, for use in a multi-policy application, to which less work has been devoted. We also assume treatment effect dynamics are homogenous across states. That is, they follow the same path over time with respect to the policy's effective date. This may not be the case if, for example, there is learning across states. Our model accommodates differences across states in a policy's substantive detail or implementation that affect the magnitude of the policy's effect, but does not allow for relative differences across states in event-time. See Sun and Abraham (2021)[4] for a more detailed discussion.

© 2023 Stein BD et al. *JAMA Health Forum.*

eReferences

1. Borusyak K, Jaravel X, Spiess J. Revisiting Event Study Designs: Robust and Efficient Estimation. Updated April 19. Accessed January 21, 2023. https://arxiv.org/abs/2108.12419
2. Goodman-Bacon A. Difference-in-differences with variation in treatment timing. *Journal of Econometrics*. Dec 2021;225(2):254-277. doi:10.1016/j.jeconom.2021.03.014
3. Schuler MS, Griffin BA, Cerdá M, McGinty EE, Stuart EA. Methodological challenges and proposed solutions for evaluating opioid policy effectiveness. *Health Services and Outcomes Research Methodology*. 2020;21(1):21-41. doi:10.1007/s10742-020-00228-2
4. Sun L, Abraham S. *Estimating Dynamic Treatment Effects in Event Studies with Heterogeneous Treatment Effects*. 2020. Accessed September 8, 2021. http://economics.mit.edu/files/14964

© 2023 Stein BD et al. *JAMA Health Forum.*
