# Supplementary material for: Association of Selected State Policies and Requirements for Buprenorphine Treatment With Per Capita Months of Treatment
Source: JAMA Health Forum. 2023 May 26;4(5):e231102. doi: 10.1001/jamahealthforum.2023.1102 (PMC10220518; doi:10.1001/jamahealthforum.2023.1102)
Supplement: Supplement 2. — Data Sharing Statement [file jamahealthforum-e231102-s002.pdf]

Data Sharing Statement

Stein. Association of Selected State Policies and Requirements for Buprenorphine Treatment With Per Capita Months of Treatment. *JAMA Health Forum*. Published May 26, 2023. doi:10.1001/jamahealthforum.2023.1102

Data
Data available: No

Additional Information
Explanation for why data not available: Data protected by a Data Use Agreement from IQVIA.
